# Investigation of the Impact of Social Vulnerability and Racial Disparity on COVID-19 Infection and Death Rates Among Georgians (USA)

**DOI:** 10.61148/2836-2810/IJEPHR

**Published:** 2025

**Authors:** Mohamed Mubasher, Lynnette Ametewee, Reinetta Thompson Waldrop, Peter Baltrus, Sabrina Mobley, Rakale C. Quarells, Michelle Nwagwu, Chanelle Harris, Kamaria Glover, Mekhi Hill, Brittany D. Taylor, Tabia Henry Akintobi

**Affiliations:** Morehouse School of Medicine.

**Keywords:** covid-19, pandemic, african americans/blacks, social vulnerability, determinants of health, health disparities, racial disparities, uninsured, georgia, community engagement, community health worker

## Abstract

**Introduction:**

The novel coronavirus (COVID-19) continues to shed light on the disproportionately negative impact of public health pandemics among racial/ethnic minorities and other systematically marginalized communities persistency experiencing poorer health and health outcomes. Far less statistical investigation has been conducted to confirm the disease agnostic social determinants correlated with the intersection of emergent crisis, chronic health conditions and local contexts to inform proactive response strategies. This study investigated the influence of Social Vulnerability (SV), barriers to access to vaccination and Racial Disparities (RD) on COVID-19 infection and death rates among Georgian residents using Georgia Department of Public Health data and County Health Rankings & Roadmaps

**Method:**

We adjusted analyses for other predictors of outcomes by using the Poisson Generalized Linear Mixed Models (with county as the unit of analysis). We iteratively modeled county-specific infection/death rates as a function of the Social Vulnerability Index (SVI, % Racial Population Gap (RPG) [(60+years % White - %African Americans/Blacks (AA)/Black)], education, %unemployed, %uninsured, % obese, % fully vaccinated, racial differences in respiratory infection discharge rates and %AA /Black residents (w/o RPG in the model).

**Results:**

Per adjusted models’ results of COVID-19 related death,, I) AA/Blacks, relative to Whites, were 51% more likely to die (p-value <0.0001), 1) by age-specific and overall estimates(p-values <0.0001 and 2) at a younger mean age (p-value < 0.0001), II) 1% increase in SVI increases the risk of death by 25% (p-value <0.0001) and III) risk of death decreases by 2.3% for every % increase in 60+ years old Whites vs. Black males county residents (p-value <0.0001). The case infection rate a) decreased by a.1) 0.1% for every percent population increase in the racial gap (i.e., more Whites than AA/Blacks in a county) (p-values = 0.0122) and a.2) 27% for every % increase of those fully vaccinated(p-value < 0.0001). The rates also increased by a) 17% with every 1% increase in SV Index p-value <0.0001) b) 1% for every 0.1% increase in those a) obese (p-value < 0.0001) and b) uninsured (p-value < 0.0001).

**Conclusions:**

Attention to the Social Vulnerability Index (SVI) factors associated with COVID-19 illness and death signal the need for proactive prevention and mitigation interventions prior to and in the wake of public health pandemics thereby bridging the health disparity gap

## Introduction:

Historically, African Americans (AA)/Blacks have had disproportionate outcomes for severity of illness and hospitalizations in the wake of public health emergencies including Severe Acute Respiratory Syndrome (SARS), the novel influenza A (H1N1), and Zika [1,2] due to factors including socio-economic vulnerabilities [3]. Predisposing factors among many AA/Blacks include living and working in overcrowded housing / multigenerational homes with lack of running water, low income status, unsecure jobs without health insurance, use of public transportation, all of which hinder optimal mitigation of the risks of infectious diseases [4,5]. Across the nation, the novel coronavirus (COVID-19) continues to shed light on how such factors increase exposures due to the inability to practice optimal risk reduction behaviors including physical distancing and sheltering-in-place [6]. Higher prevalence of pre-existing health conditions such as obesity, asthma, hypertension, and diabetes, that increase the risk of contracting COVID-19 and suffering from more severe COVID-19 related outcomes further exacerbate this issue, thereby resulting in disproportionate hospitalization, illness and death [7,8,9].

Studies in several states have reported higher numbers of COVID-19 cases, hospitalizations, and deaths among minorities compared to white populations [10, 11, 12]. In Georgia (GA), AA/Blacks represent 32% of the population and constituted 83% of COVID-19-related hospitalizations [13]. In Louisiana and Chicago, AA/Blacks account for only one third of the states’ populations yet, at the time of this report, represent approximately 70% of COVID-19 mortalities [12, 13].

Similarly, public health authorities indicate that the elderly population, those over 60 years of age, are at highest risk for developing and dying from COVID-19 [13, 14]. Data from the JHU COVID-19 tracking database indicated that while more than 60 percent of the COVID-19 cases are among people over 60, there were differences by age group and across cities and states [14]. According to the Centers for Disease Control and Prevention (CDC), 8 out of 10 deaths from COVID-19 have been in people 65 years and older (CDC, 2020, February 11). However, few studies have investigated outcomes for older AA/Blacks [15]. A study of patients in California showed that AA/Blacks ages 65 and over died twice as often as their white counterparts and younger AA/Blacks died 2.5 times as often as their white counterparts [16].

As the pandemic unfolded, a focus on socio-political barriers such as lack of health insurance, and the policies that engender inequities in access to care, emerged. The COVID-19 pandemic called attention to the fact that non-elderly AA/Blacks, aged 19 to 64, are overrepresented in service industry jobs and disproportionately make up “essential workers” such as retail grocery workers, nursing home aides, health-care workers and custodial staff [17,18]. Although employed, many of these individuals are uninsured because they cannot afford their share of premiums for employer-based insurance. The US (health) uninsured rate among AA/Blacks aged 19 to 64 in the United States rose from 10.2% in the year 2021 to 15% in the year 2023- [19].

When addressing the uninsured, research generally focuses on traditional at-risk, and vulnerable populations. However, it is important to note that this pandemic has created a new group of uninsured, at risk individuals. Galvin (2020) reports that as of late April 2020, more than 10 million adults in the United States lost their health insurance, either due to job losses or cancelling coverage due to cost [20]. Collins et al. (2020) report that 21 million people were unemployed, by May 2020, due to the pandemic, with millions of them losing their employer-based health insurance coverage [21]. As the pandemic continues to hinder our ability to open up the economy, many of these individuals could remain out of work, and millions may not return to the workforce at all. This means people who lose their jobs and health coverage during the pandemic who live in states that have not expanded Medicaid coverage may also be less likely to gain insurance through that public program [20, 21, 22]. This will increase the number of uninsured individuals that must be considered, as social policies related to health insurance coverage, particularly during a pandemic, are re-examined.

Living in a county in GA as an AA/Black seems to pose a significant risk for COVID-19 infection. It has been shown by another independent study of county data from April 2021, prior to GA’s reopening that the percentage of AA/Black residents was independently associated with higher rates of COVID-19 cases [23]).

The purpose of this research is to investigate the impact of social vulnerability and racial disparity on COVID-19 infection and associated fatality rates among hospitalized GA patients aged 60 years and over. Concomitantly, we also aim to determine the sociopolitical determinants that create barriers to health care access, and the public health implications of a disparate program for access to vaccines. Our analyses examine individual-level COVID-19 death data that makes county level COVID-19 case and death rates. This analysis examines data approximately two and a half months after GA’ s reopening and includes COVID-19 related deaths, as well as cases.

## Materials and Methods:

### Study Measures:

**Figure 1.**
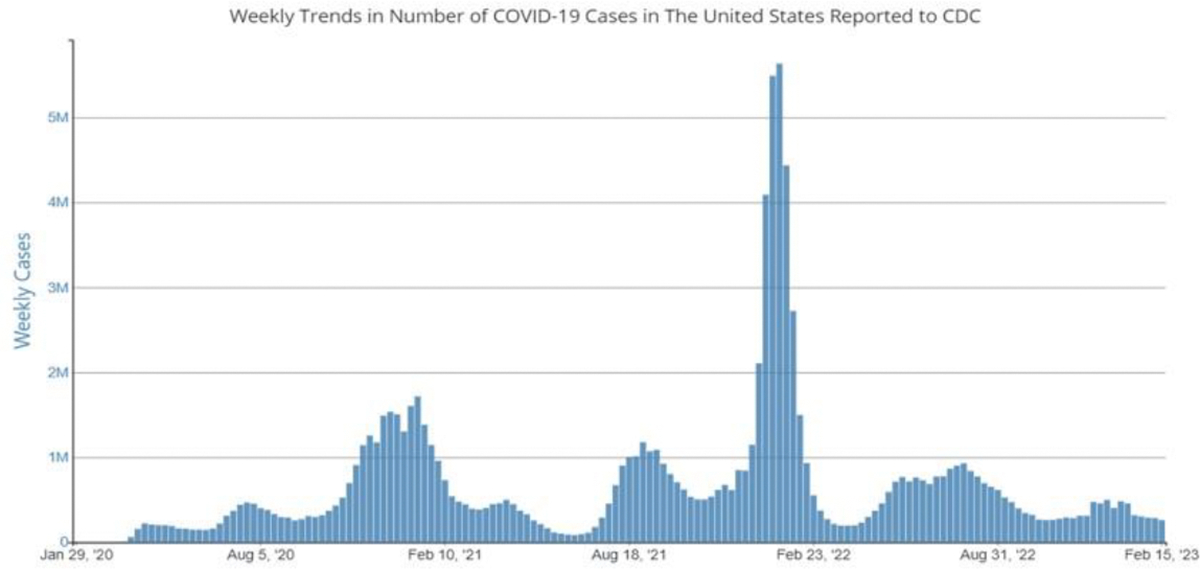
[24].

We purposely selected March 3, 2022, as a cutoff date for data analyses to allow for measuring the impact of social vulnerability while the COVID-19 cases where surging among US population as detailed in Figure 1 [24]. To our determination and according to John Hopkins reports, COVID-19 infection and mortality started tapering off/declining towards the end of Spring, 2022 [24]. Our intent was to cutoff our data for analyses to emphasize the devastating effects of the pandemic that was mediated by the impact of racial disparity prior to the decline in COVID-19 infection and death rates. County-and patient-specific data were sourced from the COVID-19 Status Report [25], the Online Analytical Statistical System [26], the Agency for Toxic Substances and Disease Registry (ATSDR) at CDC [27] and the University of Wisconsin Population Health Institute and Robert Wood Johnson Program County Health Rankings [28]. The unit of analysis in this report was GA counties (n=159). The outcome variables of interest were COVID-19 infection and death counts (for summary descriptive results), infection, and death rates (for statistical modeling results). Chronic conditions, which is an explanatory/mediator variable, included chronic lung disease, diabetes mellitus, cardiovascular disease, chronic renal disease, chronic liver disease, immunocompromised condition, neurologic/neurodevelopmental condition, pregnancy and other [26].

The Social Vulnerability Index (SVI) was utilized to assess social and political determinants of health hypothesized to influence differences in prioritized outcomes. Developed by ATSDR at CDC and according to its developers [27] “this index is a way to combine the factors that may make a community more vulnerable and less likely to recover easily from a natural disaster into a single index” [27]. “The basic formula projects how corresponding disaster affiliated outcomes may unfold is:

Risk = Hazard * (Vulnerability − Resources, where Risk is the likelihood or expectation of loss; Hazard is a condition posing the threat of harm; Vulnerability is the extent to which persons or things are likely to be affected; and Resources are those assets in place that will diminish the effects of hazards” [29].

**Figure 2:**
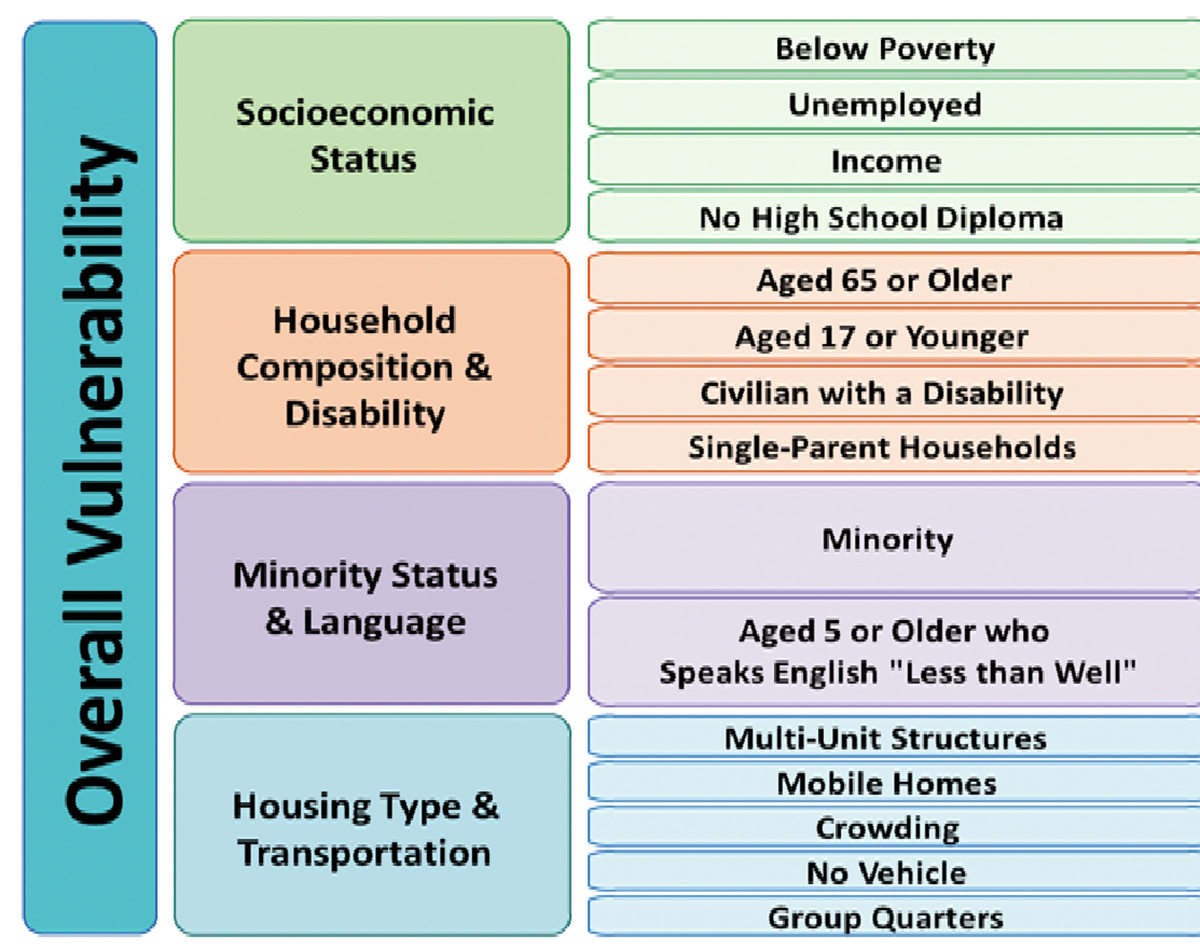
Domains and Constructs of Social Vulnerability Index [3, 26].

The SVI considers 15 census derived variables across four domains including socioeconomic status, household composition, minority status and language and housing type and transportation (Figure 2) [27]. To construct the SVI, each of the 15 census variables, except per capita income, was ranked from highest to lowest across all census tracts in the United States with a non-zero population (N = 65,081). Per capita income was ranked from lowest to highest. A percentile rank was then calculated for each census tract for each of these variables. Percentile ranks were calculated using the formula:

PercentileRank=(Rank-1)/(N-1) where N = the total number of data points, and all sequences of ties are assigned the smallest of the corresponding ranks. In addition, a tract-level percentile rank was calculated for each of the four domains based on an across-the-board sum of the percentile ranks of the variables comprising that domain. Finally, an overall percentile rank for each tract was calculated as the sum of the domain percentile rankings. This process of percentile ranking—for all variables, for each domain, and for an overall SVI— was then repeated for the individual states. In our analyses, we used the proportion ≥ 60 years residents in each census-tract as a weight to obtain county-specific SVI using the formula:

county-specificSVI=∑overallcounty'scensustractsof[cenus-tract-secificproportion>60yearsresidentsmultipliedbycenus-tract-specificSVI].¯

Using GA Department of Public Health and CDC data, we cross-sectionally modeled county-specific frequency of COVID-19 between February 2020 and March 3, 2022, as a function of SVI, comorbid conditions, obesity, county-specific residential racial gap and insurance status.

Recognizing that SVI formulation comprised racial/ethnic minority group status, when we ran “Poisson Regression“ models with SVI wee only included factors that were not explicitly accounted for by SVI. These factors included sex, the county-specific % difference between Whites and AA/Blacks, and residents 40 years or older. The county-specific health/health care indicators included % obese, % with health insurance, % vaccinated, and % county difference between Whites and AA/Blacks in respiratory discharge rates.. These variables were introduced in series of iterative statistical models that did not include SVI. Additionally, all model(s) (explanatory) terms were screened for presence of pairwise or joint correlation to avoid having highly correlated variables in the same model. In selecting these explanatory variables, we focused on AA/Blacks and Whites 60+ years of age since they represent the age groups that have been among the most vulnerable to COVID-19 infection and death [14] at the time of this study. Primary, county-specific covariates of interest were difference in % population (county residents) between Whites and AA/Blacks (% Whites - % AA/Blacks) [30] which we termed % population racial gap, the % AA/Blacks males/females county residents and % difference between AA/Blacks and Whites in pulmonary infection hospital discharge. Other potentially mediating/adjusting covariates of interest were % with no health insurance (uninsured), % unemployed, % with college/higher degree and % adults with obesity. Our adult obesity measure was based on estimates from the CDC Interactive Diabetes Atlas (2016) (using Behavioral Risk Factor Surveillance Survey, [31,32]) and defined as the percentage of adults (20 years and over) reported Body Mass Index greater than or equal to 30 kg/m2. The uninsured measure was defined as the percentage of the population under 65 without health insurance, using estimates from the Small Area Health Insurance (2017) (County Health Rankings and Roadmaps, 2020c). The education measure for “some college” was defined as the % adults ages 25 – 44 years with some post-secondary education and is estimated from the American Community Survey (2014 – 2018) (County Health Rankings and Roadmaps, 2020d). The unemployment measure was defined as the % population ages 16 and older unemployed but seeking employment and is based on 2018 estimates from the Current Population Survey, Current Employment Statistics, and the Unemployment Insurance System (County Health Rankings and Roadmaps, 2020e). We also added a covariate of county-specific % full vaccination (CDC and Georgia Department of Public Health) utilizing data from March 2020 to March 3, 2022.

### Steps undertaken to retrieve data from sources and compose for analyses

Analyses used GA county-level data (n=159 counties) for COVID-19 death and infection rates as primary outcome measures up to March 3, 2022. We also included county-specific variables from other sources, listed below, that potentially serve as covariates/independent/predictive (of the outcomes) factors. demographics (age groups, sex, ethnicity) % difference between Whites and AA/Blacks 60+ years old residents % unemployed; % with college/higher education health/health care indicators: % obese, % without health insurance, % vaccinated, % county difference between Whites and AA/Blacks in respiratory discharge rates and, Social Vulnerability Index.

We present below the algorithm we followed in retrieving data from various sources, detailed below, concatenating and/or merging it to form several county-specific data sets (n= 59, each) for data summaries, univariate and multivariate statistical analyses. The GA Department of Public Health COVID-19 statistics (https://dph.georgia.gov/covid-19-status-report) allowed for access to the daily cumulative COVID-19 death/infection statistics per county stratified by age, sex, race/ethnicity and chronic conditions (yes/no for any of diabetes, chronic lung disease, cardiovascular disease, chronic renal/liver disease, immunocompromised condition, neurodevelopmental condition). We consequently tallied these deaths/infection counts by county and grouped them by age groups (40 to <60, 60 to <65, 65 to < 75 and >= 75 years), race (White, AA/Blacks, others), sex (females/males) and chronic conditions(yes/no). This data is then stored in a data file (call it file “A”, which has n=159 (number of counties in GA) data points).

The GA Department of Public Health county population statistics allowed the download of county-specific variables. (https://oasis.state.ga.us/oasis/webquery/qryPopulation.aspx) Specifically, population counts of residents were stratified by age, sex, and race. These counts constitute the denominator for calculating the proportion/rate of infection/ death (also) stratified by age, sex and race. Other variable downloaded are below. Respiratory discharge rates stratified by the groups described above The % difference in county residents between White and AA/Blacks age 60+ years % AA /Black males and females.

We stored this data in another file (call it file “B”, which has also n=159 data points) The Robert Wood Johnson data sources(https://safesupportivelearning.ed.gov/resources/robert-wood-johnson-foundation-county-health-ranking).facilitated the download of GA county-specific data on % obese % with health insurance % unemployed % with college/higher education This data was then stored in a data file. Call this file “C” which has also n=159 data Points. D. Centers for Disease Control and Prevention (https://www.atsdr.cdc.gov/placeandhealth/svi/index.html facilitated access to the GA census tract-specific SVI which we pooled over GA counties using weighted average by % 60+ years residents.

This data was then stored in a file (“ D” (with n=159)) We merged files A, B, C and D by county to form our univariate as well as multivariate analyses -ready data set.

### 2.3 Statistical Analsyses Methods:

We summarized data using mean/median and standard deviation for continuous variables and frequency and percentages for categorical ones. We also performed exploratory analyses using correlation methods (e.g., SVI versus infection and or death rates) and graphics to pave the way for our models that primary used the methodology of the Generalized Linear Mixed Models (GLMM) [33]. The GLMM used Poisson distribution with a log of counts as the link function additional to the log of population counts at risk as an offset to iteratively model the outcome variables, as a function of the study covariates. We evaluated “overdispersion” in our Poisson models and whenever necessary we adjusted the covariance matrix of the model with the chi-square statistic divided by its degrees of freedom as a scaling factor. Separate models were fit to circumvent having highly correlated covariates in the same model (e.g., unemployment and % with college degree or higher). Main effects and interaction models were explored to determine the best fit of the functional relationship between the infection/death rates and the covariates. We conducted Receiver Operating Characteristics (ROC) Analyses to attempt determining which of the covariates where more important in predicting infection and death rates due to COVID-19. To that effect we used a Generalized Linear Mixed Model (GLMM) with county having a random intercept while regressing death/cases as a function of each covariate. Subsequently we used a logistic function model with thepredictaed probabilities obatined by the GLMM to determine the ROC probabilities.

## Results:

### Overall :

Table 1.0 summarizes the distribution of the study outcomes and covariates. By March 3, 2022, the GA county-specific data (N=159 counties) estimated an average of 10947.7 COVID-19 case rate (cases per 100,000) (SD: 3683.013; min, max: 5,143.85, 43130) and 280.89 COVID-19-related death rate (deaths per 100,000) (SD: 122.57; min, max: 80.34, 854.39) (Table 1). This data displays a significant variability and gap among counties, in both infection and death rates.

By March 3, 2022, there were 1,913,823 confirmed COVID-19 cases and 29,991 deaths in GA. Among cases, 28.51% were AA/Blacks, 45.69% were Whites, 12.62% from other ethnicities and 9.94% were Hispanic/Latino. Of the 29,991 deaths, 33.84% were AA/Blacks, 61.37% where White and 5.22% were of Hispanic/Latino origin.

### Exploratory/Univariate County-Specific SVI, Racial Population Gap (RPG), Adults Obesity And COVID-19-Age of Death Results:

**Figure 3:**
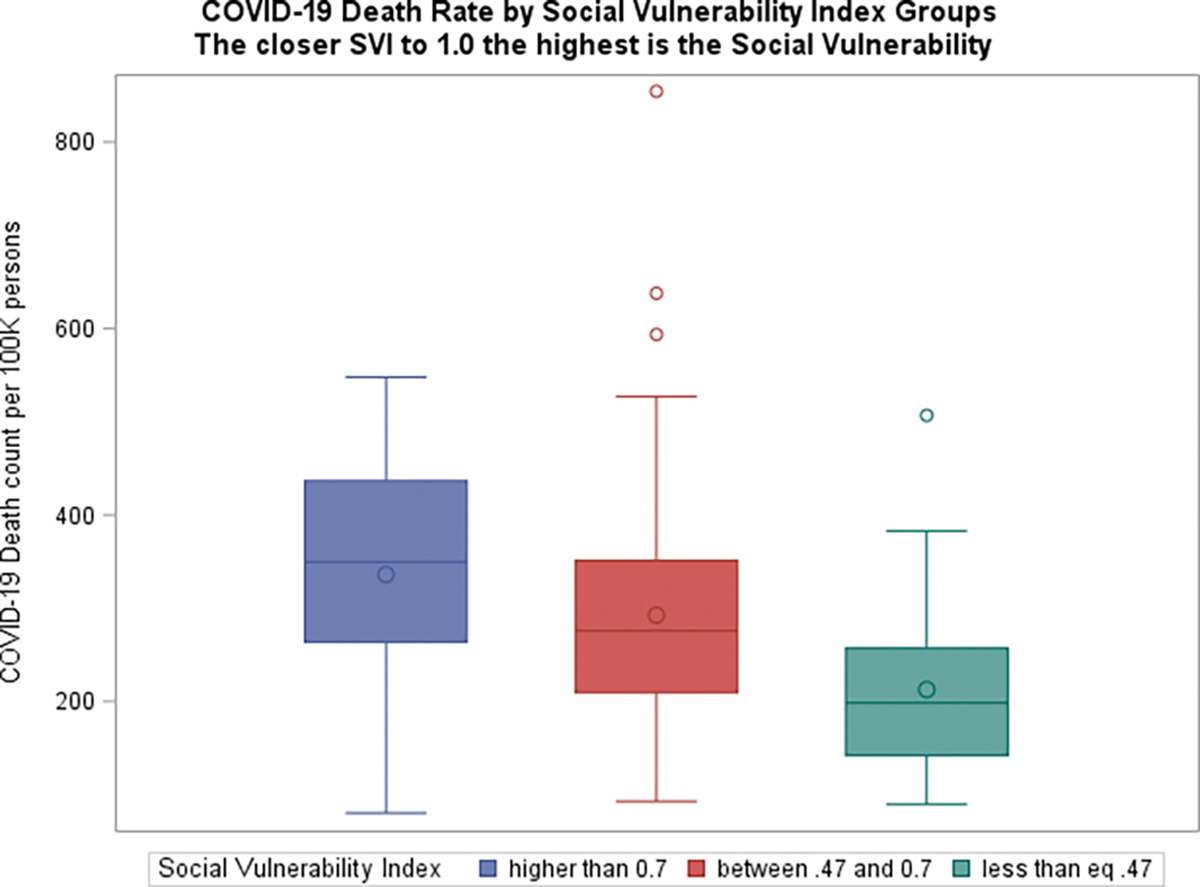


**Figure 4:**
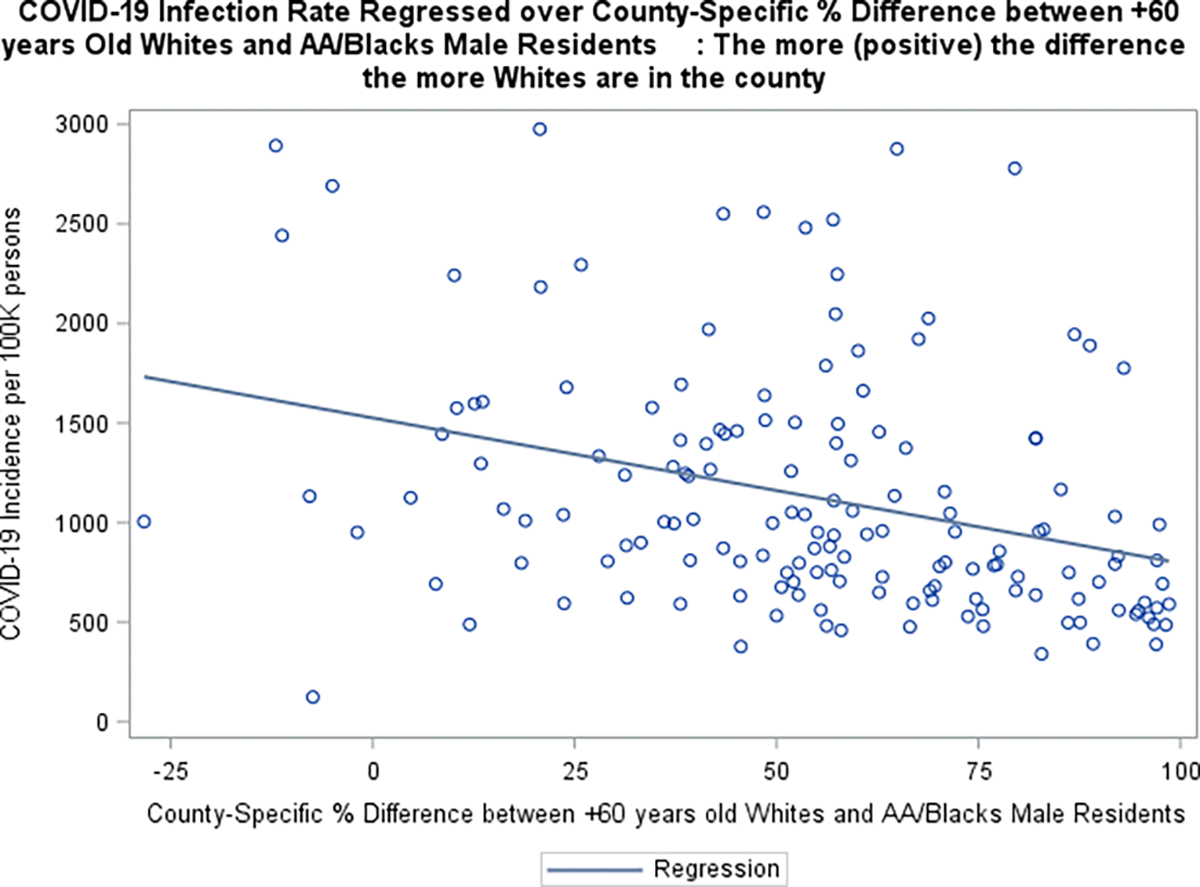


**Figure 5:**
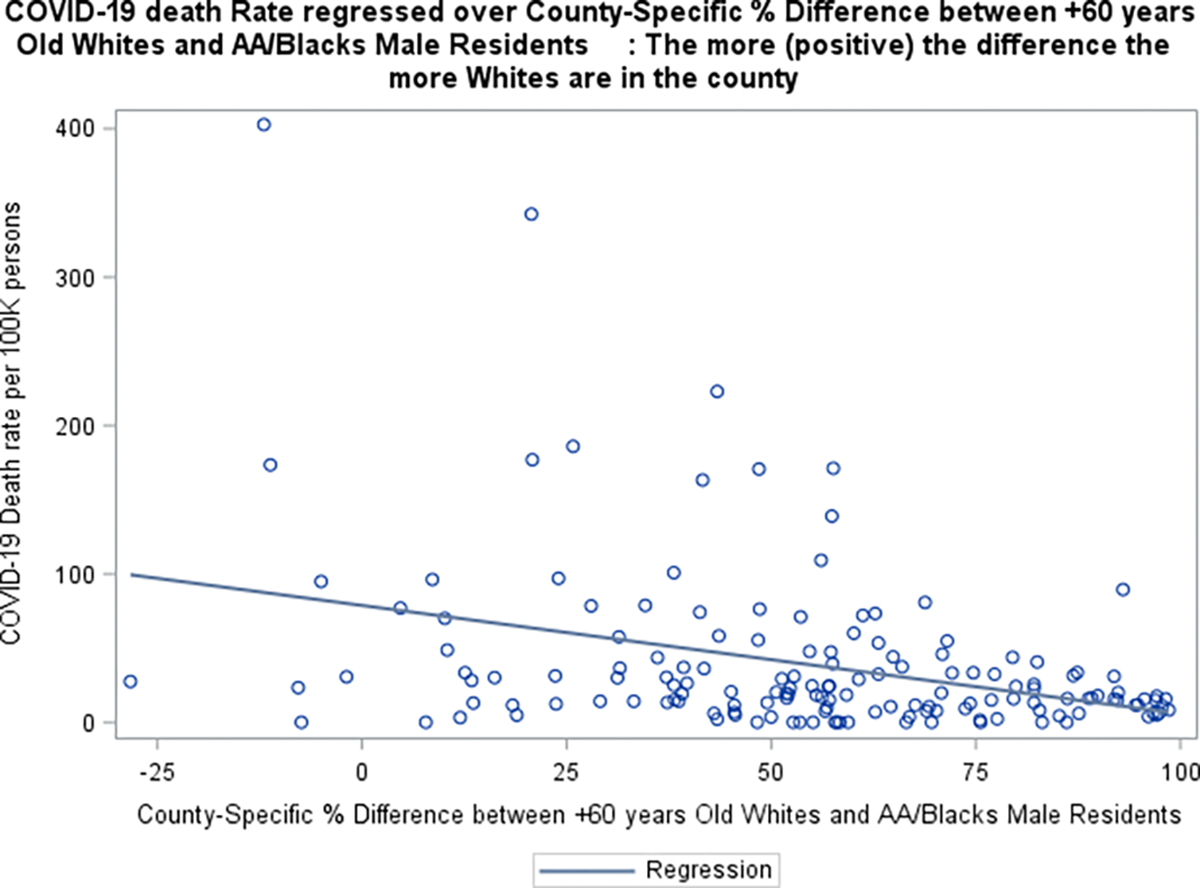


**Figure 6:**
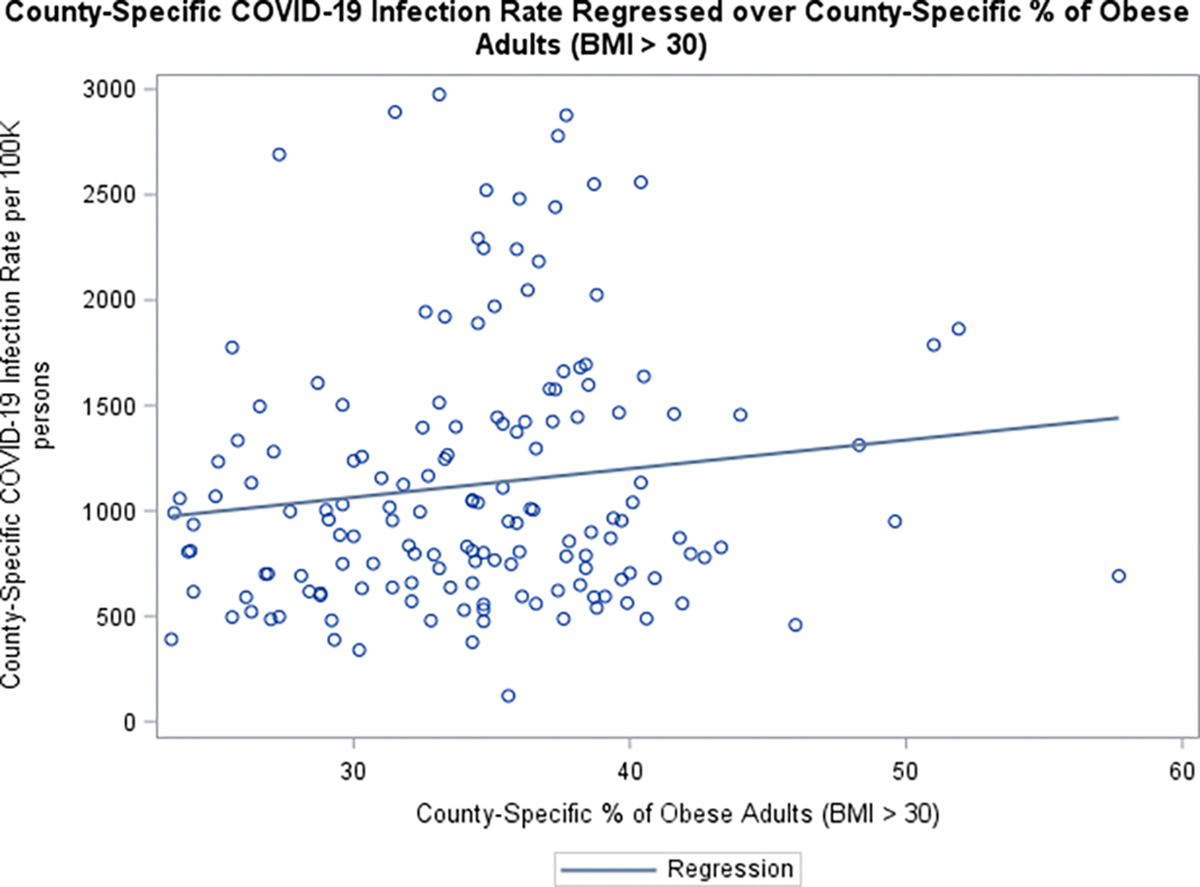


Boxplots graphs of COVID-19 related death against SVI (grouped using 30^th^ and 60^th^ percentiles of the (SVI) data), suggested that counties with higher social vulnerability are more likely to have higher death rates (Figure 3). Additionally, COVID-19 infection /death data also suggested that counties with more % 60+ years old Whites than AA/Blacks seem to have lower rates. (Figures 4–5). Furthermore, the raw data also suggested that counties with more % obese adults seem to have higher COVID-19 infection rates (Figure 6).

**Figure 7:**
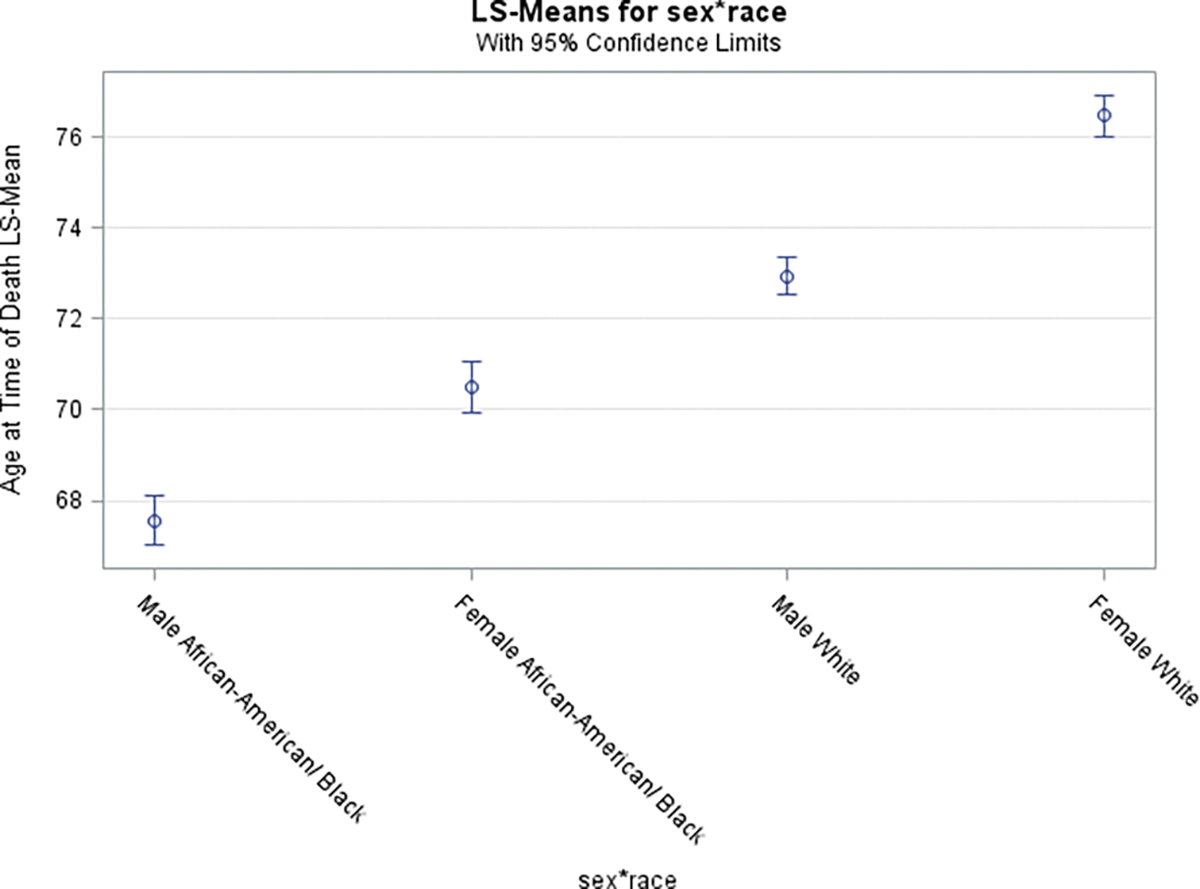


To estimate adjusted age of COVID-19 related death, we fitted a regression model with age/Sex/chronic conditions/race-stratified GA county-specific COVID-19 death data. This revealed that AA/Black males and females when compared to their White counterparts died at significantly younger age (p-value =0.02) (Figure 7)

### Adjusted findings:

Risk factors for death due to COVID-19 Per Poisson gender and chronic conditions- adjusted models, age-specific death incidence ratios display that AA/Blacks, relative to Whites, were 35% to 72% more likely to die of COVID-19 (Table 2.0). They also, irrespective of gender or presence of chronic conditions, died at a younger mean age (years) relative to Whites [(AA/Blacks (males with (without) chronic conditions): 68.57 (67.48) vs. Whites (74.32 (72.87); AA/Blacks (females with (without) chronic conditions): 70.8770.73) vs. Whites (77.12 (76.56)); p-values < 0.001].

Adjusted iterative independence (no interaction terms) Poisson models further estimated a significantly increasing (in age) excess of death due to COVID-19 for individuals 60 years or older compared to those younger than 60 (3.2 to 40.9 fold increase, p-value <0.0001) (Table 2.1). The models also indicated that male gender (Incidence Rate Ratio (IRR)=1.45, p-value <0.0001), AA/Blacks race (IRR=1.51, p-value <0.0001), increased SVI (p-value<0.0001) and chronic conditions (IRR=1.38, p-value<0.0001) are all independent significant risk factors for death due to COVID-19 (Table 2.1)

## Discussion

Results of these analysis signal distinct contextual factors, beyond those of the individual, significantly associated with COVID-19 mortality. Adjusted loglinear/ Poisson models indicated that, AA/Blacks relative to Whites were 40% to 86% more likely to die of COVID-19. They died at a mean younger age relative to Whites. Analyses also estimated a 1% significant decrease in case rate for every percent population increase in the racial gap (i.e., more Whites than AA/Blacks in a county, p-values = 0.0122) and a significant 59% increase in death rate for AA/Blacks relative to Whites. Analyses also estimated a significant, 17% increase in cases rate with every percent increase in social vulnerability, a 1% increase for every percent increase in obesity and uninsured (respectively) and a significant 27% decrease for every percent increase of those fully vaccinate. Additionally, it is also useful to note that the loglinear/Poisson models estimated a 25% increase in the risk/likelihood of death due to COVID-19 for every percent increase in SVI. Though this estimate is cross-sectional in nature and emanates from comparing counties with a % differential in SVI, it is important to recognize that SVI explicitly as well as implicitly incorporates **actual census data** that closely pertains to adverse social determinants like a household being below poverty level, unemployment/no high school education of the head of the household as well as poor/crowded housing conditions, lack of transportation means and household including members with disabilities. Therefore, it is not at all surprising, in our opinion, that a modest increase in SVI translates into potentially aggravating deterioration in social determinants. Such deterioration is closely aligned with racial disparities and adverse inflation in COVID-19 infection and death rates for underserved communities. It is also interesting to note that Reciever Operating Characterestics (ROC) analyses suggested that counties with higher percentage of residents older than 75 years of age, Black/AA and of higher SVI were among the most at risk of COVID-19 death. On the otherhand, it was also deduced from the results that counties with lower percentage of fully vaccinated residents, higher SVI and higher percentage of black/AA residents were among the most at risk of COVID-19 infection

The individual level COVID-19 data also yielded some noteworthy results. People with chronic conditions died at an older age than those without chronic conditions. This may be partially due to the fact that chronic conditions usually develop later in life [34]. However, it highlights the fact that people who are younger and healthy (in terms of not having a chronic condition) are still vulnerable to death associated with COVID-19 infection. The racial age difference in age of death was quite substantial, with AA/Blacks dying at younger ages than Whites in all gender and chronic disease categories. This is quite alarming as it might have been hypothesized that any observed race difference in age at death would be due to AA/Blacks suffering from chronic diseases at earlier ages than Whites. Some of the differences may be due to race differences in non-diagnosed disease and conditions among those without a chronic disease, or due to race differences in disease severity among those with a chronic condition. The reasons for these large race differences in age of death merit further investigation.

The county level ecologic analysis revealed that % AA/Black continued to be a substantial predictor of COVID cases and fatality rates two and half months past reopening the economy (May-June 2020). While Gaglioti et.al. (2020) found a similar association prior to GA re-opening; the virus continued to spread with a surge in rates in recent weeks. It was anticipated that, perhaps, the association with % Black would have weakened [36]. A strength of our analysis was the inclusion of a chronic disease rate and obesity rate variable. The race difference in COVID-19 outcomes persisted even after taking into account the racial gap in respiratory infections hospital admissions and percent obesity, as well as their socioeconomic indicators.

For decades, AA/Blacks have traditionally been uninsured at rates higher than their white counterparts. Using a 3-year average from 2003 to 2005, DeNavas-Walt, et al. (2010) found the uninsured rate for AA/Blacks to be 19.5% compared to 11.2% for non-Hispanic Whites [37] Buchmueller, et al. (2016) determined that in 2013, AA/Blacks were uninsured at a rate of 25.8% compared to 14.8% for Whites [37]. Cohen, R.A. & Terlizzi, (2020) report that in 2019, the uninsured rate nationally for AA/Blacks was 13.6%, compared to 9.8% for non-Hispanic Whites [39]. The Patient Protection and Affordable Care Act (ACA) of 2010 closed the gap between uninsured AA/Blacks and non-Hispanic Whites, particularly in states that expanded Medicaid. In states that chose to provide health insurance through Medicaid Expansion programs, those social policy decisions decreased the uninsured rate for AA/Blacks 11.4 percentage points from 21.5% in 2013 to 10.1% in 2018. However, in non-Medicaid Expansion states the uninsured rate for AA/Blacks for the same period decreased by only 8.6 percentage points from 27.3% to 18.7% [39]. GA is one of fourteen states, the majority of which are in the south, that has not implemented a Medicaid Expansion program. In 2018, the uninsured rate for AA/Blacks in GA was 15% [20], 1.4 percentage points higher than the national average.

Health insurance and the vaccination are central to increasing access to care and reducing virus mitigation. While these may be a catalyst for those who are ready to engage in the healthcare systems and to be immunized, there is still a justified history of distrust of both the health care system, further exacerbated by the new and unknown territory related to the pandemic. There are still a number of community health communication issues to address culturally-bound stigma, misinformation and related beliefs associated with immunization in general, and the no-man’s land associated with COVID-19 among AA/Blacks. Some AA/Blacks think they are immune from contracting the virus because they are protected by their ancestors. Further, and notwithstanding the results of this report, young AA/Blacks are now being diagnosed with COVID-19 at increasing rates [17] but believe that they can’t contract COVID-19. We must both respect and combat mass media and socially normed beliefs that can foster myths and mistrust among marginalized communities.

The health communication strategies or interventions designed to promote testing of complications associated with infections in health care systems should include the strategic engagement community health workers (CHW). According to the American Public Health Association (APHA), a CHW is a “frontline public health worker who is a trusted member of and/or has an unusually close understanding of the community served. This unique position, skill and community trust enables the worker to serve as a liaison/link/intermediary between health/social services and the community to facilitate access to services and improve the quality and cultural competence of service delivery” [40]. Their deployment through research, health, policy and environmental health strategies among vulnerable populations are central to community navigation of health information as well as testing strategies designed to mitigate the virus and that is developed to address the unique concerns, priorities and perceptions of AA/Blacks, across the life course.

We found large inter-county differences for COVID-19 positive cases which could be due to underreporting or lack of health infrastructure for testing in counties with no/less cases. Our study showed a broad range from 0 to <12,000 cases by county. Therefore, further research is needed to understand whether the low number of cases in some counties is a true reflection of disease rates, or due to a lack of testing infrastructure, underreporting of cases or other contextual factors that cannot be elucidated by secondary data analyses, alone. Harvard Global Health Institute conducted an analysis for National Public Health Radio, using a Vulnerability Index (VI), to COVID-19, for all US states based on several factors including prevalence of comorbid conditions, % minority, population density, % with health insurance, poverty and healthy food availability [41]. The VI along with longitudinal tracking of the pandemic, other policy decisions and availability of contact tracing were used to develop, for each US state, two distinct estimates for number of daily testing needed (per 100k population) to mitigate and suppress the pandemic. If it is found that some counties are truly exceptional in their low numbers/lack of cases further demonstrating the need for further analysis (ideally longitudinal and mixed methods) should be conducted to determine what characteristics make these counties “positive deviants”.

Research on cardiovascular disease in AA/Blacks has found communities at low risk in Atlanta [42], with factors such as neighborhood and psychosocial characteristics of the residents being related to resilience or better health outcomes [43]. A qualitative assessment through an environmental scan and key informant interviews may uncover factors that serve as barriers or facilitators associated with risk for the comorbidities associated with COVID-19. They include but are not limited to food deserts/availability of healthy foods and safe places to engage in physical activity [23]. This rigorous, community engaged investigation may also elucidate other contextual and environmental protective factors that have not been previously correlated to risk and resilience due to the untapped research territory presented through the COVID-19 pandemic.

### Strengths and Limitations:

The unit of analysis in our work was the county (n=159). In pursuing the Poisson regression models, we calculated the actual gender-specific differences (gap) between AA/Blacks and Whites 60+ years of age in county residents as well as the differences in respiratory infection discharge rates. We directly infused these differences in model fitting iterations as covariates/ predictors of outcomes. This methodology, to our knowledge, was not previously investigated and pursued in racial disparity statistical analyses which we hope will be further investigated in future studies. The Poisson models also rendered a highly satisfactory fit of the functional relationships between infection/death rates and the study covariates.

The ecologic nature of the data potentially subjects the findings to the ecological fallacy, whereby the significant associations found may not be valid at the individual level. In the case of AA/Blacks percent this is not a concern as it has already been well established that AA/Blacks suffer from worse COVID-19 related outcomes than Whites [13]. Insurance and employment status should be examined at the individual level to determine if there are associations with the outcomes hold at that level of analysis. Because of the lack of race-specific data at the county level we were only to do the analysis on the rates for the entire population. When the data becomes available the analysis should be repeated to see if the associations with the outcomes are of equal magnitude across race-ethnicity groups.

Percent uninsured estimates used for analyses [28] only included those who were 65 years of age or younger and our analysis focused on 60+ of age population. This may have led to underestimating the impact of lack of health insurance as a mediator and/or effect modifier of COVID-19 infection and fatality rates.

Essential next steps to instigate public health response, policy and action will be the coupling the characterization of counties with high vulnerability, COVID-19 case and death rates with multilevel intervention strategies that bring together diverse stakeholder (policy, community, clinical and public health, among others). Initiatives funded by the National Institutes of Health, the Centers for Disease Control and Prevention and the Office of Minority Health are currently leveraging the expertise of the co-authors of this report, with strategic partnerships to promote linkages to vaccination uptake, social services and clinical care in order to address pandemic-instigated needs as well as preexisting, obstinate individual- and context-level risk factors [44–46].

## Conclusions:

The Coronavirus-19 pandemic continues to foster conversations around health and healthcare reform. Central to these discussions and associated action strategies will be public health researchers, practitioners, community residents affected and those in public health policy. Given the presence of significant racial disparities and social vulnerability that exasperated the adverse impact of the pandemic among racial minorities, further targeted policies are needed to fundamentally address these gaps. Further longitudinal research studies with adequately powered studies are needed to investigate the variance in COVID-19 cases and deaths across GA counties. The disproportionately large racial/ethnic differences in age of death merit further investigation to understand why and how some communities are resilient or may be characterized by protective factors in the face of social vulnerability and social or political determinants that statistically forecast poor outcomes. In the interim, the realities of the increased COVID-19 morbidity and mortality among older AA/Blacks call for mixed-method strategies that are community-engaged, policy informed and with demonstrable impact. Results will require strategies meeting at the intersection between systems change, research or evaluation rigor and public health practice designed to advance health equity.

## Figures and Tables

**Figure 8.1:**
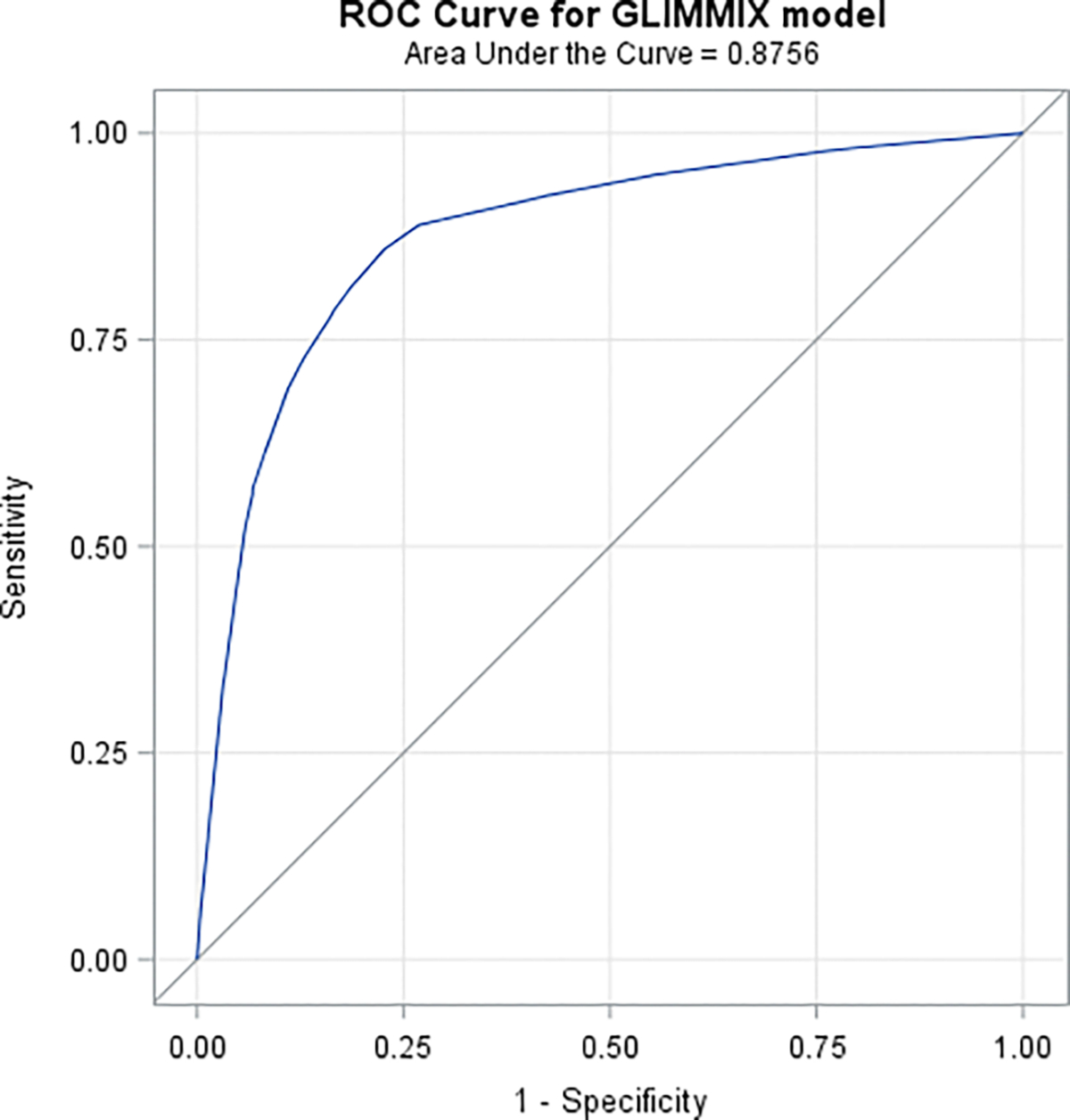
Receiver Operating Characteristics (ROC) When regressing death as a function of Race

**Figure 8.2:**
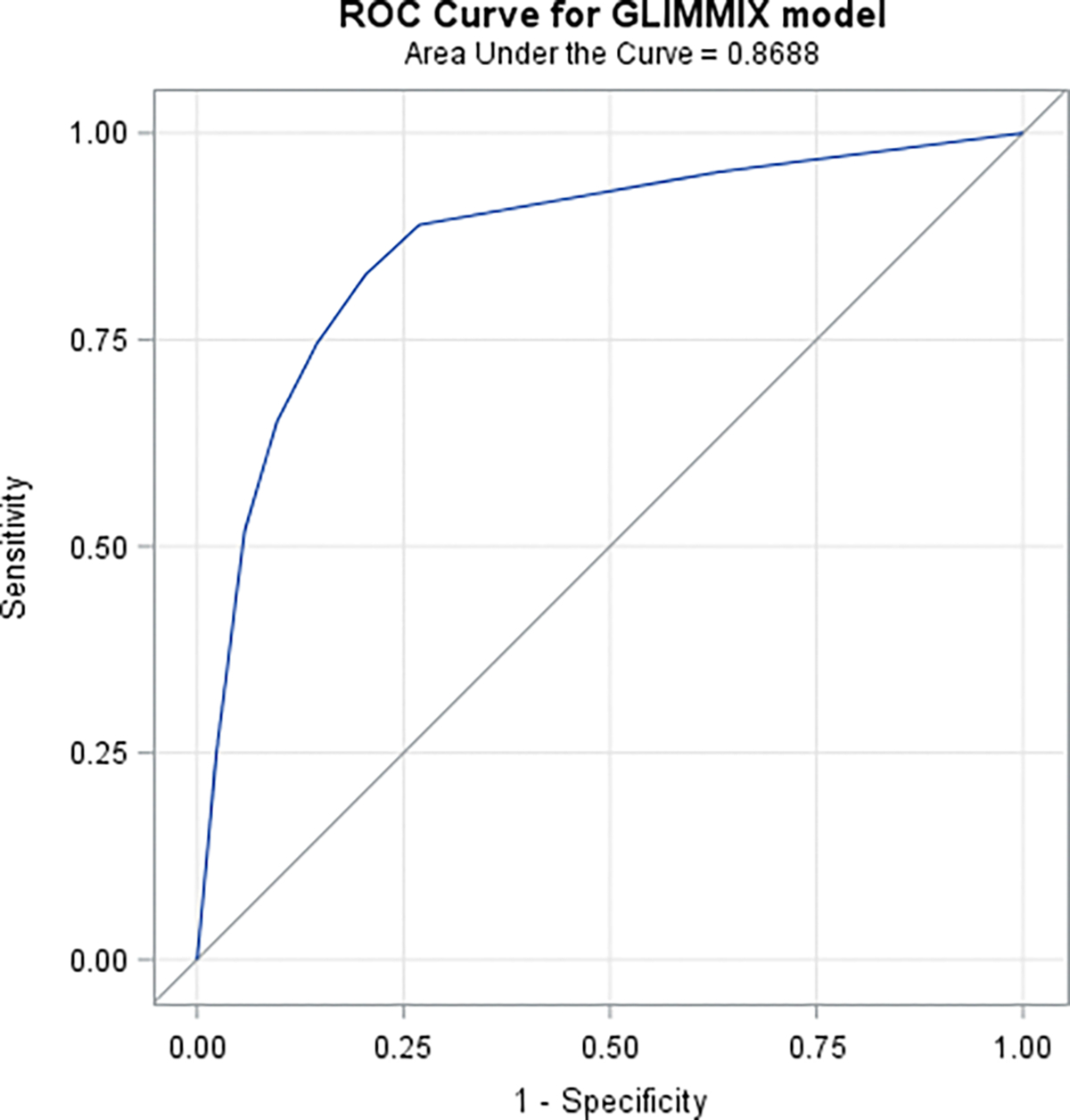
Receiver Operating Characteristics (ROC) When regressing death as a function of Age

**Table 1.0:** Overall Distribution of Georgia County-Specific (N=159) COVID-19 Outcomes and Covariates.

	Mean	SD	Median	Min	Max
**Social Vulnerability Index (SVI)**	0.57	0.17	0.58	0.16	0.93
**Population**	64764.1	139981.7	22550.0	1665	1.02
**Number of People Fully Vaccinated**	24837.2	64883.26	7169.0	547	489811
**Number of People Vaccinated with at least One Dose**	27673.8	72528.36	8015.0	620	549659
**Proportion vaccinated with at least one dose**	0.32	0.07	0.32	0.15	0.53
**COVID-19 Case rate (per 100,000)**	10947.7	3683.03	10366.1	5144	43130
**COVID-19 Death rate (per 100,000)**	280.89	122.57	270.3	80	854
**COVID-19 14-day case rate**	432.83	271.96	399.3	64	2235
**20^th^ Percentile Income**	18946.2	6596.98	16756.0	5756	46694
**80^th^ Percentile Income**	90086.9	20825.62	84685.0	57480	183293
**% Adults with Obesity**	34.45	5.89	34.7	23	58
**% Physically Inactive**	30.85	5.45	30.6	19	50
**% Current Smokers**	18.26	2.30	18.2	13	24
**% With Some College or higher**	50.72	11.96	49.7	21	82
**% Unemployed**	4.44	0.92	4.2	3	8
**% With No Health Insurance**	16.54	2.62	16.3	10	28
**% With Access to Exercise Opportunities**	54.17	26.79	59.3	0	100
**Antigen cases**	2146.71	3490.10	1088.0	39	23571
**County-specific difference (White -AA/Black) in Respiratory conditions Discharge rate**	2.01	2.89	1.7	−7	12
**County-specific difference (White -AA/ Black) in % Female residents**	49.06	30.65	51.0	−35	98
**County-specific difference (White -AA/Black) in % Male residents**	54.11	28.08	56.6	−28	99
**Hospitalization rate (per 100,000 residents)**	491.75	958.15	204.0	10	6637
**County-specific % AA/Black Respiratory Conditions discharge rate**	8.02	2.95	7.7	0	19
**County-specific % Whites Respiratory Conditions discharge rate**	10.11	2.29	9.6	4	19
**County-specific % AA/Black Females +60 years old**	24.34	15.30	23.4	0	64
**County-specific % AA/Black Males +60 years old**	21.79	14.01	20.7	0	60
**County-specific % White Females +60 years old**	73.40	15.39	74.7	29	99
**County-specific % White Males +60 years old**	75.91	14.11	77.2	31	99

**Table 2.0:** Poisson Regression Results of Comparison of Age-Adjusted COVID-19 Death Rates Between African American/ Black vs. White (Adjusted for Sex, Chronic Conditions and Full Vaccination
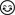

African American/ Black vs. White					
Age (years)	Log of Death Count	Standard Error	Incidence of Death Rate Ratio	Excess in Risk of Death	P-Value
**< 60**	0.4820	0.1009	1.62	62%	<.0001
**60 TO < 65**	0.5015	0.1366	1.65	65%	0.0002
**65 TO < 75**	0.5402	0.08818	1.72	72%	<.0001
**>75**	0.3004	0.06470	1.35	35%	<.0001

**Table 2.1:** Poisson-Regression-Adjusted Estimates of the Magnitude and Corresponding Significance of the Association between Death due to COVID-19 and Age, Sex, Ethnicity, and Presence of Chronic Conditions (Before Adjusting for Fully Vaccinated Individuals).

Effects/Comparisons	Log of Death Counts	Standard Error	Incidence Rate Ratio	P-value	Interpretation (Fold increase in the risk of death for AA/Blacks vs. Whites in the corresponding effect category)
**60 TO < 65 VS. < 60 years**	1.1825	0.0886	3.26	<0.0001	3.36 times
**65 TO < 75 VS. < 60 years**	2.4326	0.0698	11.39	<0.0001	11.39 times
**Older than 75 VS. < 60 years**	3.7114	0.0616	40.91	<0.0001	40.9 times
**Males vs. Females**	0.3752	0.0435	1.45	<0.0001	1.45 times
**AA/ Black Vs. White**	0.4131	0.0457	1.51	<0.0001	1.51 times
**Chronic Conditions: Yes vs. No**	0.3256	0.0438	1.38	<0.0001	1.38 times
**Social Vulnerability Index**	0.2231	0.004	1.25	<0.0001	1.25 times (25% for each unit Increase in SVI)
**Difference (White-AA/Black) in % County Residents (Males)**	−0.02223	0.004856	0.973	0.0001	2.3% decrease for White vs. Black County residents

**Table 3:** Receiver Operating Characteristics (ROC) When using a Generalized Linear Mixed Model to regress death/cases as a function of each covariate

ROC Model	Area	Standard Error
**COVID-19 Death as a function of Race**	0.8756	0.00126
**COVID-19 Death as a function of older age**	0.8688	0.00133
**COVID-19 Death as a function of Chronic Conditions**	0.6465	0.00158
**COVID-19 Death as a function of SVI**	0.6154	0.00197
**COVID-19 Infection as a function of Full vaccination**	0.6374	0.00231
**COVID-19 Infection as a function of SVI**	0.6109	0.001878
**COVID-19 Infection as a function of Race**	0.6089	0.00231

## Data Availability

Data associated with this study was publicly accessible.
